# Inflammatory Modifications in Paranasal Sinuses and Ostiomeatal Complex Anatomical Variations in Jet Aircraft Pilots: A Computed Tomography Study

**DOI:** 10.1055/s-0043-1773761

**Published:** 2023-11-29

**Authors:** Yeda da Silva, Luciana Munhoz, José Rodrigues Parga Filho, Andreza Gomes Damasceno, Cesar Felipe França da Rosa, Eduardo Bilaqui Zukovski, Erik Zhu Teng, Emiko Saito Arita, Cláudio Campi de Castro

**Affiliations:** 1 School of Medicine, Institute of Radiology, Universidade de São Paulo, São Paulo, SP, Brazil; 2 Department of Stomatology, School of Dentistry, Universidade de São Paulo, São Paulo, SP, Brazil; 3 Pinhais Medicine School, Pinhais, Paraná, PR, Brazil; 4 Natal Air Base, Estrada para Aeroporto SN, Parnamirim, RN, Brazil; 5 School of Medicine, University of Buenos Aires, Buenos Aires, Argentina; 6 School of Medicine, Faculdade Pequeno Príncipe, Curitiba, PR, Brazil

**Keywords:** multidetector computed tomography, diagnostic imaging, maxillary sinus, aerospace medicine, aviation

## Abstract

**Introduction** Jet aircraft pilots are exposed to huge pressure variation during flight, which affect physiological functions as systems, such as the respiratory system.

**Objectives** The objective of the present investigation was to evaluate inflammatory changes of paranasal sinuses of jet aircraft pilots before and after a jet aircraft training program, using multislice computed tomography (CT), in comparison with a group of nonairborne individuals with the same age, sex, and physical health conditions. A second objective of the present study was to assess the association between the ostiomeatal complex obstruction and its anatomical variations.

**Methods** The study group consisted of 15 jet aircraft pilots participating in the training program. The control group consisted of 41 nonairborne young adults. The 15 fighter pilots were evaluated before initiating the training program and after their final approval for the presence of inflammatory paranasal sinus disease. The ostiomeatal complex anatomical variations and obstructions were analyzed in pilots after the training program.

**Results** Jet aircraft pilots presented higher incidence of mucosal thickening in maxillary sinus and anterior ethmoid cells than controls. Prominent ethmoidal bulla showed significant association with obstruction of the osteomeatal complex.

**Conclusions** Jet aircraft pilots present increased inflammatory disease when compared with nonairborne individuals. The presence of a prominent ethmoidal bulla is associated with ostiomeatal complex obstruction.

## Introduction

The paranasal sinuses are complex structures comprising a group of connected bone cavities designed as the maxillary sinuses, the frontal sinus, the sphenoid sinuses, and ethmoid cells. 1 They have multiple functions for the respiratory system, such as to humidify the nasal cavity, thermal insulation, and moistening of the inspired air. 2 Additionally, they act absorbing the impacts to cranial structures as well as reducing the weight of the skull. 2

Inflammatory and infectious diseases are relatively frequent in the worldwide population 3 and exhibit a wide range of symptoms and imaging features, from asymptomatic cases with incidental imaging findings 4 to spread infection in adjacent structures into the face and head, with life-threatening complications. 3

It has been documented that some populations have higher risk to the development of inflammatory sinus disease, such as patients with cystic fibrosis, 5 acute optical neuritis, 6 inflammatory bowel disease, 7 coal workers, 8 or the presence of maxillary teeth roots within the maxillary sinus. 9

Previously, it was reported that jet aircraft pilots have volume alterations in maxillary sinus, due to the exposition of diverse occupational hazards, such as high altitude and sudden movements, and multiple repetitive exposition to high gravitational force (Gz) changes. 10 However, the evaluation of paranasal sinuses for detecting inflammatory disease has not been studied in jet aircraft pilots.

Besides the volume alteration in maxillary sinuses, preceding observations on facial modifications in jet aircraft pilots throughout their careers have been observed by the Brazilian Air Force physician and imaging radiologist, who is also the first author of this study and has been working at the Brazilian Air Force for 13 years. The physician noticed the ostiomeatal complex may be also affected.

The ostiomeatal complex is an area lateral to the middle turbinate in which the frontal and maxillary sinuses drain, as well as the anterior and medium ethmoid cells. It is constituted by the ethmoidal infundibulum, the uncinated process, the ethmoid bulla, the semilunar hiatus, and the middle meatus. 11

Thus, the aim of this investigation was to evaluate inflammatory changes of paranasal sinuses of jet aircraft pilots before and after a jet aircraft training program, using multislice computed tomography (CT), in comparison with a group of nonairborne individuals with the same age, sex, and physical health conditions. The second aim of the present study was to assess the association between the ostiomeatal complex obstruction and its anatomical variations.

## Methods

The present prospective study was conducted between March (initial CT examination) and November (final CT examination), 2015. Sample size was determined according to the number of fighter pilots of the Natal Air Base selected to the jet aircraft training program in the same year. The approval of the ethics committee was obtained in the Medicine School Faculty, in the session of 08/26/2015 (No. 351/15). The guidelines of Helsinki were followed. All participants signed the written informed consent form previously established by the Ethics Committee.

### Characteristics of the Aircraft. Pilots and Control Group

The jet aircraft used in the training is the A-29 (the Super Tucano version for the Brazilian Air Force), which is a single-engine, stepped-tandem, multi-purpose military turboprop aircraft.

The study population consisted of 18 pilots of the A-29 Super Tucano fighter of the Joker squadron 2 ^nd^ /5 ^th^ , aged 23 to 27 years old, in the city of Natal, state of Rio Grande do Norte, with no associated comorbidities. The training program period is 8 months.

The control group consisted of young adults, with the same age as the pilots (20 to 30 years old), male, healthy, without previous comorbidities.

The study and follow-up of the paranasal sinuses tomography was performed in the city of Curitiba, state of Paraná, Brazil. Considering the control group, initially, the study had a total of 60 volunteer participants who accepted to participate in the project. It was explained that all participants would have to repeat tomography of the paranasal sinuses in a period of 8 months in the city of Curitiba. In this follow-up of 8 months, there were 15 withdrawals and the exclusion of 3 participants who underwent upper molar tooth removal surgery, with a total of 41 people in the control group at the end of the study.

### Inclusion and Exclusion Criteria

As an inclusion criterion, only healthy candidates were admitted, and they were previously tested for satisfactory general health, which also considered absence of smoking and drinking habits. Moreover, teeth removal before or during the training program and sinonasal surgery, such as maxillary sinus graft or surgery intervention for any inflammatory disease history were considered as exclusion criteria.

Acute airway infection, episodes of headache, and barotrauma during training program were also collected.

### Imaging Parameters, Acquisition, Evaluation and Data Collection

The CT scans were performed on the Toshiba Aquilion (Toshiba Activion, Medical Systems Corporation, Japan) 16-channel multislice CT equipment. The slice thickness was 3 mm, with 4 mm interval, field of view (FOV) of 180 mm, 120 kV, 150 mA, slice time of 1 second, soft tissue and bone filter, window with 2000 HU level and opening of 200 HU. This same protocol was always applied for all imaging exams.

The fighter pilots and the control group had images collected at 2 different times: before training and after the 8-month training period. The tomographic examinations of the pilots were performed after a period of at least 10 hours after the flight practice. Subsequently, the examinations were analyzed by two experienced radiologists.

**Fig. 1:**
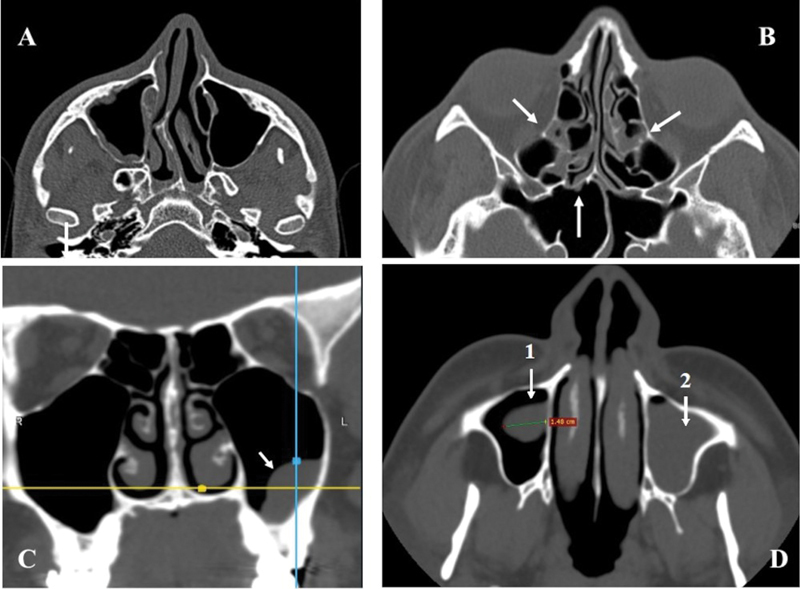
( **A** ) Mucosal thickening: Axial CT section (bone window) showing mucous thickening of the right maxillary sinus and deviation of the nasal septum to the right; ( **B** ) Mucosal thickening: Axial CT section (bone window) showing mucosal thickening of the left sphenoid and anterior and posterior ethmoidal cells (arrows); note the septum deviation on A and B. ( **C** ) Pseudocyst of mucous retention in the left maxillary sinus seen on CT (bone window) in the coronal plane (arrow). ( **D** ) Axial section on CT (bone window). In the right side a measurement of polyp (1). In the left side, total opacification (2).

The presence of inflammatory tomographic changes observed in the all the paranasal sinuses were then classified into 2 categories, according to the typical cases observed in the sample, based on the previously described literature 2 : (1) Thickening of the sinus mucosa (> 2 mm) ( Fig. 1A and B ); (2) antral polyps and pseudocysts (mucous retention cysts) ( Fig. 1C and 1D . respectively); and (3) opacification ( Fig. 1D ).

**Fig. 2:**
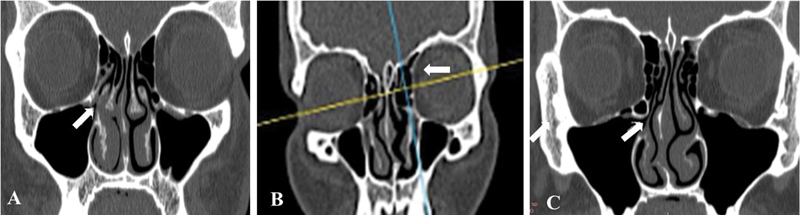
( **A** ) Coronal section on CT (bone window) showing obstruction of the right ostiomeatal complex (arrow). ( **B** ) Coronal slice (bone window) showing Agger nasi cell on the left (arrow)t; ( **C** ) Coronal section (bone window) showing Haller cell on the right, promoting narrowing of the ostiomeatal complex (arrow).

**Fig. 3:**
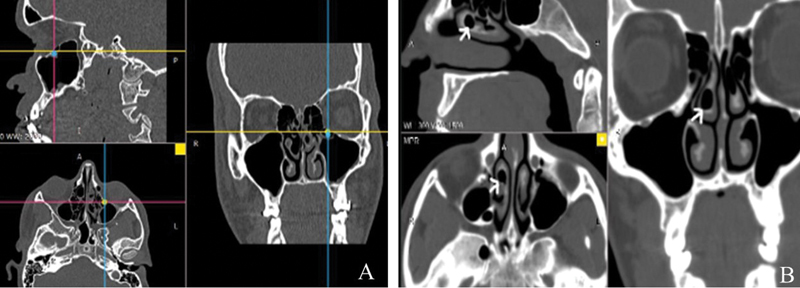
( **A** ) Prominent ethmoidal bulla on the left (bone window) in the orthogonal sagittal, axial, and coronal planes (arrows). ( **B** ) Bullous nasal turbinate on the right (tip of the white arrow) shown on CT (bone window).

**Fig. 4:**
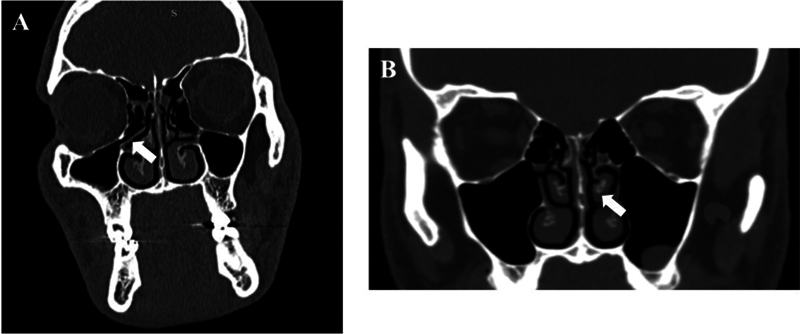
( **A** ) Coronal section (bone window) showing medial deviation of the uncinate process on the right; ( **B** ): Coronal section (bone window) showing polyp/pseudocyst of mucous retention in the left maxillary sinus and paradoxical curvature of the middle turbinate on the left (white arrow).

For the jet aircraft pilots only, considering the ostiomeatal complex, initially, the presence of its obstruction was recorded ( Fig. 2A ), then variants were assessed and associated with obstruction of the osteomeatal complex, after the training program. The variations were classified as: (1) Agger nasi cells ( Fig. 2B ); (2) Haller cells ( Fig. 2B ); (3) septum deviation; (4) prominent ethmoidal bulla ( Fig. 3A ); (5) bullous nasal turbinate or bullous shell ( Fig. 3B ); (6) paradoxical middle turbinate ( Fig. 4A ); and (7) changes in the uncinate process, such as hypertrophy, medial deviation, and horizontalization ( Fig. 4B ).

The computed tomography images were evaluated by two observers at the workstation using the OsiriX DICOM Viewer software (PIXMEO SARL, Geneva, Switzerland), and the tomographic findings were confirmed in the axial, sagittal, and coronal planes.

### Statistical Assessment

The results of quantitative variables were demonstrated by medians, minimum and maximum values. Qualitative variables were described as absolute numbers. Data regarding the health and working conditions of the pilots was also characterized.

The Wilcoxson and the Mann-Whitney nonparametric tests were applied to verify differences between groups. The Fisher exact test was applied to assess associations between variables.

The data were assessed using Stata/SE v.14.1 (StataCorpLP, College Station. TX, USA), and statistical significance was considered when *p* < 0.05.

## Results

### Descriptive Statistics Regarding the Jet Aircraft Pilots Working and Health Conditions

Cabin mean altitude of 6,577 feet (±880); the flight mean altitude was 8,142 feet (±892); and the total flight hours was 93 hours (±15). Two pilots reported headache (13.3%); 4 reported airway infection (26.7%); and there were 2 cases of barotrauma (13.3%).

### Mucosal Thickening and Presence of Polyps/Pseudocysts in Comparison with Control Group

Jet aircraft pilots presenting an increase in the mucosal thickening in maxillary sinuses and anterior ethmoidal cells were observed in the group of pilots after the training program ( *p* = 0.01 for the right maxillary sinus; *p* = 0.03 for the left maxillary sinus; and *p* = 0.04 for the anterior ethmoidal cells). When the pilots were compared with the control group, mucosal thickening was increased in the maxillary sinuses of pilots ( *p* = 0.05 for right maxillary sinus; p ≤ 0.00 for left maxillary sinus). No statistically significant differences were noticed for the presence of polyps and pseudocysts. Detailed information is available on Table 1 .

**Table 1:** Analysis of mucosal thickening and polyp/pseudocyst of mucosal retention of the paranasal sinuses (initial x final) in the pilot group and in the control group

Variable	Side	Evaluation	Group		p-value** (pilots x control)
			Pilots ( *n* = 15)	Control ( *n* = 41)	
**Maxillary sinus mucous thickening**	Right	*Initial*	*0 (0–8)*	*0 (0–15)*	*0.050*
		*Final*	*6 (0–12)*	*2.1 (0–21)*	
		p*(initial x final)	*0.018*	*0.295*	
	Left	*initial*	*0 (0–13)*	*0 (0–11)*	*<0.001*
		*final*	*10 (0–26)*	*0.5 (0–11)*	
		p*(initial x final)	*0.003*	*0.191*	
**Frontal sinus mucous thickening**	Right	*initial*	*0 (0–0)*	*0 (0–7.6)*	*0.215*
		*final*	*0 (0–0)*	*0 (0–7.8)*	
		p*(initial x final)	*-*	*0.176*	
	Left	*initial*	*0 (0–0)*	*0 (0–12)*	*0.616*
		*final*	*0 (0–4)*	*0 (0–12)*	
		p*(initial x final)	*0.317*	*0.128*	
**Sphenoid sinus mucous thickening**	Right	*initial*	*0 (0–4.7)*	*0 (0–7.7)*	*0.205*
		*final*	*0 (0–7.1)*	*0 (0–5.7)*	
		p*(initial x final)	*0.109*	*0.889*	
	Left	*initial*	*0 (0–0)*	*0 (0–12)*	*0.400*
		*final*	*0 (0–0)*	*0 (0–12)*	
		p*(initial x final)	*-*	*0.138*	
**Mucous thickening of ethmoidal cells**	Anterior	*initial*	*0 (0–7)*	*0 (0–6.2)*	*0.139*
		*final*	*0 (0–7)*	*0 (0–7.2)*	
		p*(initial x final)	*0.042*	*0.460*	
	Posterior	*initial*	*0 (0–0)*	*0 (0–8)*	*0.657*
		*final*	*0 (0–2.6)*	*0 (0–7.5)*	
		p*(initial x final)	*0.180*	*0.583*	
		*initial*	*0 (0–7.9)*	*0 (0–27)*	
**Polyp/pseudocyst maxillary sinus mucous retention**	Right	*final*	*0 (0–14.9)*	*0 (0–21)*	*0.359*
		p*(initial x final)	*0.285*	*0.700*	
		*initial*	*0 (0–21.5)*	*0 (0–18)*	
	Left	*final*	*0 (0–18)*	*0 (0–23)*	*0.814*
		p*(initial x final)	*0.715*	*0.116*	
		*initial*	*0 (0–0)*	*0 (0–7)*	
**Polyp/pseudocyst frontal sinus mucous retention**	Left	*final*	*0 (0–0)*	*0 (0–7)*	*0.545*
		p*(initial x final)	*-*	*0.317*	
		*initial*	*0 (0–0)*	*0 (0–6.3)*	
**Polyp/pseudocyst sphenoid sinus mucous retention**	Right	*final*	*0 (0–0)*	*0 (0–6.7)*	*0.898*
		p*(initial x final)	*-*	*-*	

Legend: * according to Wilcoxon test; ** According to Mann-Whitney test. Significant if *p* < 0.05.

### Ostiomeatal Complex Obstruction and Anatomical Variations in Jet Aircraft Pilots after the Training Program

The main anatomical variants of the ostiomeatal complex were: Agger Nasi cells, bullous middle concha, paradoxical curvature, deviation of the nasal septum, Haller cells, and changes in the uncinate process. The results showed that among the main anatomical variables, septal deviation was verified mostly with obstructions of the ostiomeatal complex; however, without statistically significant results, as described on Table 2 .

**Table 2:** Association of ostiomeatal obstruction and variations in jet aircraft pilots after training program, right and left sides

Variables			Right ostiomeatal complex obstruction		p-value*
			No	Yes	
**Final Septal Deviation**	Right Side	**No**	25	4	0.50
		**Yes**	21	6	
	Left Side	**No**	25	3	0.46
		**Yes**	21	5	
**Paradoxical curvature of the middle turbinate**	Right Side	**No**	38	8	1.00
		**Yes**	8	2	
	Left Side	**No**	47	8	1.00
		**Yes**	1	0	
**Bullous medium turbinate**	Right Side	**No**	35	6	0.43
		**Yes**	11	4	
	Left Side	**No**	41	4	0.04
		**Yes**	7	4	
**Haller cell**	Right Side	**No**	45	8	0.08
		**Yes**	1	2	
	Left Side	**No**	47	7	0.27
		**Yes**	1	1	
**Prominent ethmoidal bulla**	Right Side	**No**	40	8	0.62
		**Yes**	6	2	
	Left Side	**No**	43	5	0.08
		**Yes**	5	3	
**Changes to the uncinated process**	Right Side	**No**	39	10	0.33
		**Yes**	7	0	
	Left Side	**No**	44	7	0.55
		**Yes**	4	1	
**Agger nasi cell**	Right Side	**No**	25	6	1.00
		**Yes**	21	4	
	Left Side	**No**	33	5	0.70
		**Yes**	15	3	

*according to Fisher's exact test. significant if *p* < 0.05.

A statistically significant association between prominent ethmoidal bulla and obstruction of the ostiomeatal complex was observed both for the left and right sides ( *p* = 0.08 and *p* = 0.08).

## Discussion

In the present study, it was observed that there was a higher prevalence of mucosal thickening in the maxillary sinuses, followed by the anterior ethmoidal cells, after the training program in jet aircraft pilots when compared with the beginning of the program. Furthermore, when compared with the control group, pilots have more inflammatory alterations in the maxillary sinus and anterior ethmoidal cells.

Although no direct comparisons with similar studies can be performed, as this is the first investigation considering this particular sample, it was observed previously a predisposition for sinus disease in pilots with allergic rhinitis when compared with a control group; and the incidence of acute sinus disease is slightly higher in transport pilots when compared with helicopter pilots. 12

Additionally, helicopter pilots were also pointed to present a higher incidence of chronic sinusitis and pseudocysts without symptoms when compared with fighter pilots 13 ; which is slightly dissimilar from the present results, as only mucosal thickening was detected and polyps/pseudocysts did not demonstrate differences between pilots and the control group.

Possible causes for the increased incidence of inflammatory changes in pilots may be changes in cabin pressure and flight altitude, 12 13 leading to physiological changes during the flight, which seems to be a reasonable hypothesis for jet aircraft pilots as well. Moreover, reduced humidity by the flight mask may influence the results.

Regarding the anatomical variants in the ostiomeatal complex, although no statistical significance was noticed, when compared with the control group, variations were more evidenced in the jet aircraft pilots group than in the control group. The importance of the study of these anatomical variants in pilots, as well as the obstruction of ostiomeatal complex, lies in the increased inflammatory disease detected in jet aircraft pilots by their physician. It was hypothesized that the inflammatory disease in pilots could have a close influential relationship with the ostiomeatal obstruction and its variations, which could not be proven by the present research, except for the prominent (larger than usual) ethmoidal bulla.

The ethmoid bulla is the largest cell in the anterior portion of the ethmoid sinus, and eventually also denominated as “bulla bullosa” 14 . A larger ethmoidal bulla can push the middle turbinate medially, which may contact the nasal septum and osteomeatal complex obstruction, increasing the susceptibility of sinusitis development. 15

Nasal septum deviation, which was a common finding in jet aircraft pilots, is defined as the divergence between the head medium sagittal plane and the septum orientation, 16 and its prevalence in the literature ranges from 40 to 96.9% due to different criteria for the degree and morphological characteristics of septal deviation. 17

Paradoxical curvature of the medium concha has an occurrence of 7.9 18 to 25% 17 ; small medium paradoxical curvatures, which are generally bilateral, do not influence in the drainage pathways, while larger curvatures may lead to obstruction. 16

The prevalence of Haller cells or infraorbital cells is described as ethmoidal cells located nearby the orbital cavity floor, which is usually associated with the maxillary sinus roof and may influence in the mucous drainage of the maxillary sinus, leading to a predisposition to rhinosinusitis. 16 17 The Agger nasi cells are also derived from the ethmoidal cells, with an anterior positioning. 16

There is no specific investigation in the literature comparing variations in the ostiomeatal complex with obstruction of this complex, although higher opacification of the ostiomeatal complex, indicating inflammatory modifications detected by CT has already been reported when anatomical variations were present in the ostiomeatal complex . 19

The limitations of the present study were the reduced number of aspiring jet aircraft pilots in the Brazilian Air Force who join the training every year and the restricted period (8 months) of follow-up. In addition to all these limiting aspects, another difficulty was the evidence of scarcity in the literature with approaches to physiological modifications in jet aircraft pilots and similar articles for direct comparisons. Finally, future studies are needed to better understand and establish a correlation between the findings of increased inflammatory changes in the paranasal sinuses and flight variables.

## Conclusions

In conclusion, jet aircraft pilots present increased inflammatory disease when compared with nonairborne individuals. The presence of a prominent ethmoidal bulla is associated with ostiomeatal complex obstruction.
